# Enhanced Protein Separation Performance of Cellulose Acetate Membranes Modified with Covalent Organic Frameworks

**DOI:** 10.3390/membranes15030084

**Published:** 2025-03-06

**Authors:** Shurui Shao, Maoyu Liu, Baifu Tao, Kayode Hassan Lasisi, Wenqiao Meng, Xing Wu, Kaisong Zhang

**Affiliations:** 1 College of Environmental Science and Engineering, Ocean University of China, Qingdao 266100, China; shaoshurui@stu.ouc.edu.cn (S.S.); liumaoyu@stu.ouc.edu.cn (M.L.); taobaifu@stu.ouc.edu.cn (B.T.); 2023899@ouc.edu.cn (K.H.L.); 2 Key Lab of Marine Environment and Ecology, Ministry of Education, Ocean University of China, Qingdao 266100, China; 3 Key Laboratory of Urban Pollutant Conversion, Institute of Urban Environment, Chinese Academy of Sciences, Xiamen 361021, China; wqmeng@iue.ac.cn; 4 CSIRO Manufacturing, Clayton South, VIC 3169, Australia; xing.wu@csiro.au

**Keywords:** covalent organic frameworks, cellulose acetate, ultrafiltration, trade-off, protein adsorption

## Abstract

As a porous crystalline material, covalent organic frameworks (COFs) have attracted significant attention due to their extraordinary features, such as an ordered pore structure and excellent stability. Synthesized through the aldehyde amine condensation reaction, TpPa-1 COFs (Triformylphloroglucinol-p-Phenylenediamine-1 COFs) were blended with cellulose acetate (CA) to form a casting solution. The TpPa-1 COF/CA ultrafiltration membrane was then prepared using the non-solvent-induced phase inversion (NIPS) method. The influence of TpPa-1 COFs content on the hydrophilicity, stability and filtration performance of the modified membrane was studied. Due to the hydrophilic groups in TpPa-1 COFs and the network structure formed by covalent bonds, the modified CA membranes exhibited higher hydrophilicity and lower protein adsorption compared with the pristine CA membrane. The porous crystalline structure of TpPa-1 COFs increased the water permeation path in the CA membrane, improving the permeability of the modified membrane while maintaining an outstanding bovine serum albumin (BSA) rejection. Furthermore, the addition of TpPa-1 COFs reduced protein adsorption on the CA membrane and overcame the trade-off between permeability and selectivity in CA membrane bioseparation applications. This approach provides a sustainable method for enhancing membrane performance while enhancing the application of membranes in protein purification.

## 1. Introduction

Membrane technology, being a selective separation technology with various advantages such as high efficiency, low cost, simple operation and no chemical pollution, has been considered a great technique for reclaiming useful resources and removing impurities [1,2]. Ultrafiltration (UF), being an important membrane process, has been deemed effective in virus and bacteria removal, macromolecules materials/micropollutants elimination and protein concentration and purification [3,4]. As a physical method, UF can be carried out at a low temperature without chemical reaction, so it will not damage or change protein composition [5]. Therefore, UF technology is widely used in protein concentration and purification in biotechnology and the pharmaceutical industry [4].

Thousands of UF membranes with different materials and structures have been developed to date, and there has been extensive research focusing on the balance between membrane flux and selectivity (e.g., the Robeson Plot framework) or pore size optimization [6]. However, protein adsorption due to non-specific interactions between proteins and polymeric membranes remains a critical challenge. The performance of UF membranes is constrained by the inherent conflict between pore size distribution and material surface properties, leading to the undesirable adsorption of proteins on the membrane surface or within the pore channels, which will lead to decreased protein recovery and membrane fouling [7]. In order to overcome this difficulty, researchers have employed the incorporation of modifiers (polymer, nanomaterials and macromolecular additives) during membrane fabrication to mitigate the effects of protein on membranes [8,9,10]. Nanomaterials were proved to improve the resistance of membranes to protein [11,12].

Covalent organic frameworks (COFs) are a kind of crystalline nanomaterial with a topological structure formed by covalent bonding of light atoms such as C, N, O, H and B [13,14,15]. COFs possess many excellent characteristics, including orderly and adjustable pore structure, high specific surface area and adjustable organic groups, and their unique properties include high crystallinity, high porosity, low density and outstanding stability [13,14,15,16,17,18,19,20]. Meng et al. have made achievements in improving the performance of nanofiltration membranes by COF modification, including permeability, rejection and anti-fouling performance [21,22]. Jiang et al. introduced COFs into nanofiltration membranes to strengthen the desalination performance of membranes [23,24]. Lin et al. [25] incorporated sulfonated COFs into CTA/CA membranes to obtain membranes with higher permeability, selectivity and hydrophilicity. Luo et al. [26] fabricated β-cyclodextrin COF/CA membranes with excellent separation of chiral drugs, which possessed better solute flux and stronger stability. Significantly, Duong et al. [27] experimentally demonstrated that COF incorporation induces more than 50% suppression of BSA surface deposition in polymeric membrane systems.

TpPa-1 COFs can be obtained easily by the aldehyde amine condensation reaction of monomers Tp (1,3,5-triformylphloroglucinol) and Pa-1 (p-phenylenediamine) [28]. Compared with other COFs, TpPa-1 COFs are a kind of imine-based COF which are rich in functional groups containing oxygen/nitrogen, which makes them highly hydrophilic [29]. Synthesized by a combination of reversible and irreversible organic reactions, TpPa-1 COFs with a β-ketoenamine linkage exhibited exceptional crystallinity and chemical stability [30]. In addition, TpPa-1 COF nanoflowers with a petal-like structure have high specific surface area, which is beneficial to improve the efficiency of surface reactions [31,32].

Most reported UF membranes are usually fabricated using synthetic polymer materials, yet they are usually difficult to degrade, resulting in secondary pollution after use [33]. On the contrary, cellulose and its derivatives are natural polymers that represent viable alternatives, garnering global attention due to their biodegradability and environmental sustainability [34]. As one of a kind of cellulose derivatives, cellulose acetate (CA) has attracted great attention because of its great hydrophilicity, low protein adsorption, good chlorine resistance, excellent film-forming properties and relatively low cost [35,36].

The purpose of this study is, hence, to incorporate TpPa-1 COFs into a CA membrane casting solution in order to obtain a TpPa-1 COF/CA composite membrane with lower protein adsorption which breaks the permeability/selectivity trade-off effect. To achieve this, TpPa-1 COFs were synthesized via the solvothermal method and subsequently incorporated into a CA membrane doping solution as a nanomaterial to enhance its performance. The protein adsorption capability, filtration performance and stability of the fabricated membranes were examined. The results revealed that TpPa-1 COF/CA membranes have better permeability and selectivity and an enhanced anti-protein adsorption capability compared with the pristine CA membrane. This study thus demonstrates the influence of TpPa-1 COFs on membranes and provides a way to enhance the properties of CA membranes.

## 2. Materials and Methods

### 2.1. Materials and Chemicals

CA (cellulose acetate, powder, 55.0% combination of acetic acid), DMAc (N,N-dimethylacetamide, ≥99.0% (GC), MW = 87.12), Ac (acetone, ≥99.5% (GC), MW = 58.08), KBr (spectroscopically pure) and ethanol (≥99.9% (GC)) were supplied by Sinopharm Chemical Reagent Co., Ltd. Shanghai, China. Monomers Tp (1,3,5-triformylphloroglucinol, ≥97%, MW = 210.14) and Pa-1 (p-phenylenediamine, ≥99%, MW = 108.14) for the synthesis of TpPa-1 COFs were purchased from Macklin Biochemical Technology Co., Ltd. Shanghai, China. The non-woven fabric substrate (PMB-SKC) was supplied by Mitsubishi paper Co., Ltd. Japan. PEG ((Polyethylene glycol, MW = 10,000–20,000 Da) was obtained from Aladdin Biochemical Technology Co., Ltd. Shanghai, China. OVA (ovalbumin, Biotech, MW = 44.5 kDa) was bought from Macklin Biochemical Technology Co., Ltd. Shanghai, China, and BSA (bovine serum albumin, ≥98%, MW = 66 kDa) was received from Sigma-Aldrich Trading Co., Ltd. Shanghai, China. Unless otherwise specified, the water used in the experiment was deionized (DI) water.

### 2.2. Synthesis of TpPa-1 COFs

**Figure 1:**
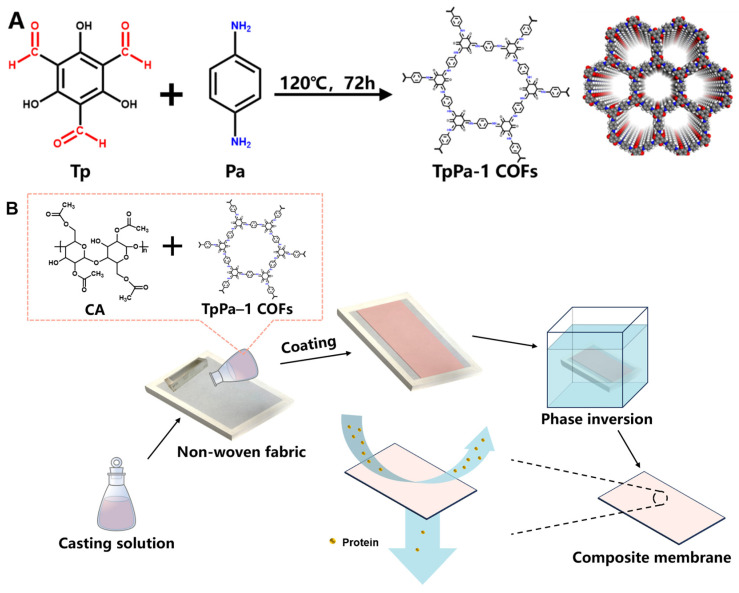
The synthetic schematic diagram of TpPa-1 COFs (**A**) and preparation process of TpPa-1 COF/CA membranes (**B**).

The TpPa-1 COFs were synthesized in accordance with the previous method reported by Meng et al. [22]. The operation referred to the method of Kandambeth et al. [28] with a few modifications. The specific process was as follows. Tp (25.2 mg, 0.12 mmol) and Pa-1 (19.2 mg, 0.18 mmol) were gently dispersed in 8 mL ethanol by ultrasound for 10 min, respectively. The dispersed solutions were poured into a Teflon autoclave and then heated at 120 °C for 72 h in an airtight nitrogen atmosphere. The filtered powder was subjected to solvent exchange with N,N-dimethylformamide and ethanol sequentially. The dry deep red powder was obtained by vacuum drying and named TpPa-1 COFs. The synthetic schematic diagram of TpPa-1 COFs is shown in Figure 1A [37].

### 2.3. Preparation of Membranes

The pristine CA and TpPa-1 COF/CA UF membranes were fabricated by the non-solvent-induced phase inversion technique. The casting solutions were conducted as presented in Table 1. Different amounts of TpPa-1 COFs were uniformly dispersed in the mixed solution of DMAc and Ac by ultrasound. Then, the same content of CA was added into the TpPa-1 COF solution and magnetically stirred at 50 °C for 12 h to obtain a homogeneous solution. Figure 1B clarified the main preparation process of M0-M5. After air bubbles were defoamed by standing, the casting solution was poured on a non-woven fabric pasted on the epoxy frame. A 200 μm-height casting knife was used to spread the casting solution horizontally. Then, the epoxy frame with casted non-woven fabric was immersed in tap water at 25 °C for phase separation. The prepared membranes were cleaned and stored in DI water for later use.

### 2.4. Characterization of TpPa-1 COFs

The morphology and elemental composition of TpPa-1 COFs were characterized by FE-SEM (Field-Emission Scanning Electron Microscope, GeminiSEM 360, ZEISS, Oberkochen, German) under 5 kV operating voltage and 10 μA operating current, and EDS (Energy Dispersive Spectrometer, Bruker, Billerica, MA, USA) under 10 kV operating voltage and 10 μA operating current. The molecular structure of TpPa-1 COFs was characterized by FTIR (Fourier Transform Infrared, ALPHA, Bruker, Billerica, MA, USA). The sample for the FTIR test was prepared as follows: 1mg of dried TpPa-1 COFs and 150 mg of KBr were mixed and ground into powder, and tested after tableting. PXRD (Powder X-ray Diffraction, D8 ADVANCE, Bruker, Billerica, MA, USA) under 40 kV voltage and 40 mA current was applied to test the crystal structure of TpPa-1 COFs. XPS (X-ray Photoelectron Spectroscopy, Axis Supra+, Shimadzu, Kyoto, Japan) was used to examine the elemental composition and content of TpPa-1 COFs. The particle size distribution of TpPa-1 COFs was characterized by a Laser Diffraction Particle Size Analyzer (LS 13 320, Beckman Coulter, Brea, CA, USA). The TpPa-1 COFs were ultrasonically dispersed in ethanol, and the upper homogeneous solution was taken for the particle size.

### 2.5. Characterization of Membranes

The top-surface and cross-sectional morphology structure of the membranes were characterized by FE-SEM at 5.0 kV. In order to enhance the conductivity of the membranes, gold was sputtered on the membrane samples before observation.

The surface roughness of the membranes was tested with AFM (Atomic Force Microscope, Cypher ES, Oxford, UK). The contact angle of M0-M5 was determined by a contact angle analyzer (DSA100, KRUSS, Hamburg, German).

The thermal stability of the membranes was evaluated by TGA (thermogravimetric analysis, TGA/DSC3+, METTLER TOLEDO, SUI, Columbus, OI, USA). The relative mass loss of the membrane samples was recorded at 30–600 °C at the heating rate of 10 °C/min in the inert gas argon atmosphere. All thermal analyses were performed on the membrane systems without non-woven fabric removal.

### 2.6. Filtration Tests of Membranes

The pure water flux and the protein (OVA and BSA) rejections were conducted to test the separation performance of membranes. A dead-end filtration system was applied to the experiment. The membrane samples were cut into 13.4 cm^2^ discs and pre-pressed at 0.15 MPa for 30 min. After the flux stabilized, the pressure was reduced to 0.1 MPa and the weight change was recorded for 20 min by SPDC data collection software connecting the computer and the digital weighing balance. The pure water flux was calculated with Equation (1):

(1) $$P=\frac{V}{At∆p}$$

where *P* (L·m^−2^·h^−1^·bar^−1^) is the pure water flux of M0-M5, *V* (L) is the volume of total infiltrated water, *A* (m^2^) is the effective area of each membrane during the filtration experiment, *t* (h) is the test time of each membrane, and ∆*p* (bar) is the transmembrane pressure.

The experiment studying protein rejection was performed using the same system. The feed liquid was replaced with 1 g·L^−1^ protein solution. The flux of the protein solution was also collected by the computer software. Each membrane was normalized through pre-compression at 0.15 MPa for at least 30 min, followed by a 10-min pre-filtration process at 0.1 MPa. A 20-min filtration test was then conducted at 0.1 MPa to collect the feed liquid and permeate liquid and to achieve a stable experimental condition. The concentrations of the feed liquid and the permeate liquid were detected by a UV spectrophotometer (759s, Lengguang, Shanghai, China). The protein rejection was calculated by Equation (2):

(2) $$R\;(\%)=(1-\frac{C_{P}}{C_{f}})\times 100$$

where *C_p_* and *C_f_* (mg·L^−1^) mean the concentration of the permeate solution and the feed solution, respectively.

The molecular weight of 90% solute trapped by the membrane is defined as the molecular weight cut off (MWCO) of the membrane [38]. The MWCO of membranes were determined by examining the rejection of 1g·L^−1^ PEG solution, and the rejection was calculated by the same equation (Equation (2)) as the protein rejection. The concentration of each solution to be measured was diluted 10 times, and then tested by a total organic carbon analyzer (TOC, TOC-LCPH, Shimadzu, Japan). The membrane pore size can be obtained by calculating the Stokes radius of PEG by Equation (3) [39]:

(3) $$a=16.73\times 10^{-12}M^{0.557}$$

where *a* (m) is the Stokes radius of PEG; *M* (Da) is the molecular weight of the solute.

In the long-term stability and anti-fouling performance test of CA membranes, the filtration experiment on M0 and M3 was carried out with DI water at 1.0 bar for 60 min, and the stable pure water flux was defined as the initial flux (*J*_0_). Then, the feed solution was replaced by 1g·L^−1^ BSA solution, and the ultrafiltration experiment was carried out for 60 min. The remaining pollutants on the membrane surface were removed with a DI water rinse. Finally, the membrane was filtered with DI water for 30 min to achieve a stable water flux (*J*). Steps 2–3 were repeated for three cycles. The anti-fouling performance of the membrane was expressed by the flux recovery rate (*FRR*), which can be calculated by Equation (4):

(4) $$FRR\left(\%\right)=\frac{J}{J_{0}}\times 100$$

### 2.7. Protein Adsorption Experiment

The membranes were cut into 2 × 2 cm^2^ and immersed in a 1 g·L^−1^ BSA solution prepared using PBS (0.1 M). The protein adsorption experiment was carried out for 24 h at room temperature. The change of BSA solution concentration before and after adsorption was examined by a UV spectrophotometer (UV2550, Shimadzu, Japan). The adsorption mass of each membrane was calculated by Equation (5):

(5) $$Q=\frac{\left(C_{0}-C\right)V}{S}\times 1000$$

where *Q* (μg·cm^−2^) is the adsorption mass of each membrane, *C*_0_ and *C* are the concentrations of BSA solution before and after adsorption, respectively, V (L) is the BSA solution volume, and *S* (cm^2^) is the area of the membrane in contact with the BSA solution.

The treated membranes were washed to remove non-adsorbed BSA protein, and thoroughly dried in an oven. The adsorption of BSA protein on the membrane surfaces was observed by a fluorescent inverted microscope (NIB900, NOVEL, Zhejiang, China).

The molecular dynamics (MD) simulation was utilized to explore the effect of TpPa-1 COFs on the protein adsorption resistance of CA membranes. The MD simulation was run on the Forcite module of the Materials Studio 2023 software package. Before conducting the MD calculations, a maximum step size of 1fs was defined, and the operation was executed in the COMPASSIII force field [40]. The geometry optimization of each system was carried out in 10,000 steps using the smart method [41]. Periodic boundary conditions were enforced in all three directions for the simulations. The MD simulation was performed in an NVT ensemble and run for a total duration of 500 ps. During the NVT simulations, the temperature of the system was maintained at 298 K by a Nose thermostat [42]. The Ewald method was employed to calculate the electrostatic energy in the system with an accuracy of 1 × 10^−5^ kcal/mol [43]. The atom-based method [43] and a cutoff distance of 12 Å were applied to calculate the van der Waals energy [44,45,46].

The cif file of the COF molecule was obtained from the Cambridge Crystallographic Data Centre (CCDC) database, while the BSA molecule was sourced from the pdb file of the Protein Database (RCSB) and its structure was simplified for simulation purposes. The specific simulation procedure is outlined as follows. The CA monomer was initially constructed and subjected to charge allocation and geometry optimization using the Forcite module. Ten optimized CA monomers were then aggregated into a single CA chain, followed by another round of geometry optimization to ensure reasonable chain conformation. The CA membrane system was constructed by inserting 10 CA chains into a simulation box using the Amorphous Cell module. For the CA/COF composite membrane system, one COF molecule was added to the box, ensuring its random distribution within the system and that this distribution maintained a three-dimensional periodicity throughout the simulation cell. Both the CA and CA/COF membrane systems underwent geometry optimization, followed by a 1 ns annealing process (298–498 K, 5 cycles, with a thermal gradient of 50 K), a 500 ps NVT ensemble dynamics simulation and a 500 ps NPT ensemble dynamics simulation.

The simplified BSA molecule was assigned charges and subjected to geometry optimization before being placed in a simulation box with the same dimensions as the CA/COF membrane. Using the Build Layer module, the BSA was positioned parallel to the membrane surface (with a 7 Å spacing), and a 20 Å vacuum layer was added above the BSA to mitigate periodic boundary effects. The BSA + CA and BSA + CA/COF systems underwent geometry optimization, followed by a 1 ns NVT ensemble simulation to achieve stable structures. Finally, a 500 ps NVT ensemble dynamics simulation was conducted to calculate the interaction energies between the BSA molecules and the membranes.

## 3. Results and Discussion

### 3.1. Characterization of TpPa-1 COFs

**Figure 2:**
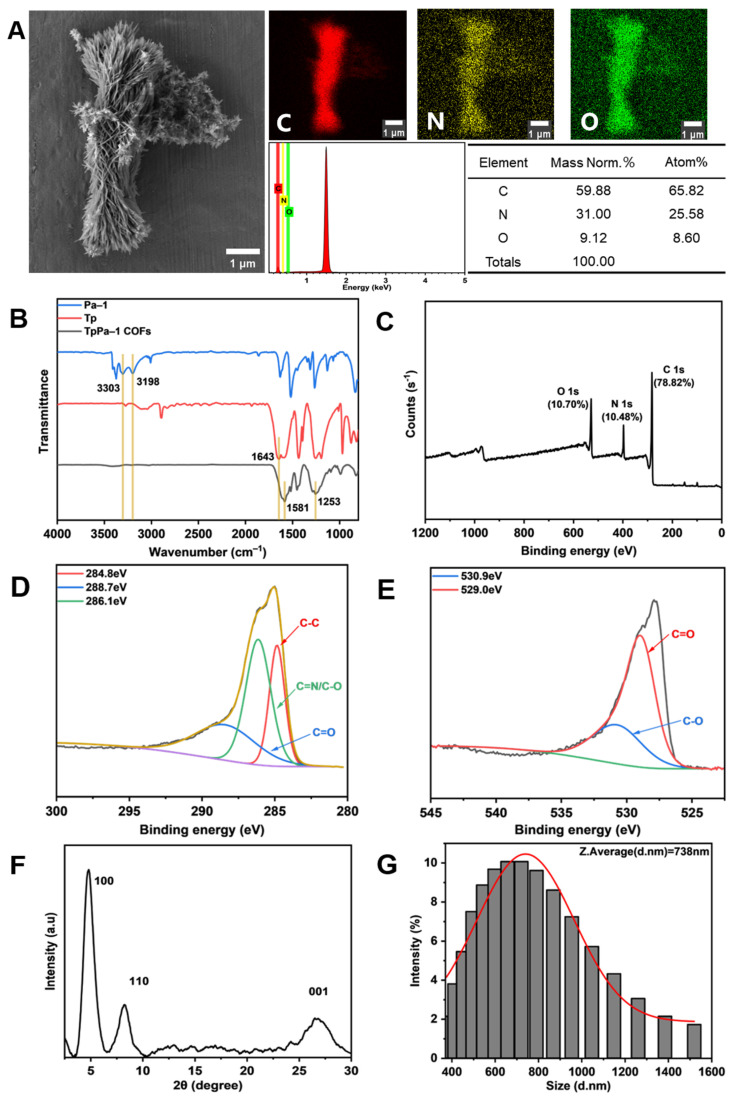
Characterization of TpPa-1 COFs: (**A**) FE-SEM and EDS; (**B**) FTIR; (**C**–**E**) XPS and the detailed spectral analysis of XPS; (**F**) XRD; (**G**) the particle size distribution.

The microstructure of TpPa-1 COFs was observed by FE-SEM. Figure 2A indicates that TpPa-1 COFs had a flower-like morphology. EDS showed that TpPa-1 COFs were mainly composed of three elements of C, N and O. The FTIR in Figure 2B explains the successful synthesis of TpPa-1 COFs. The stretching band of C=O at 1643 cm^−1^ and the NH_2_ stretching vibration bands at 3303 and 3198 cm^−1^ disappeared, and new stretching vibration bands of C=C (1581 cm^−1^) and C-N (1253 cm^−1^) appeared, which represent the complete consumption of Tp and Pa-1 and the generation of TpPa-1 COFs [37]. The XPS results (Figure 2C) demonstrated that the composition of TpPa-1 COFs was C, N and O, which illustrated that there was no deviation between the results of XPS and EDS. The TpPa-1 COFs C 1s (Figure 2D) are deconvoluted into three peaks with binding energies of 284.8, 286.1 and 288.7 eV, corresponding to C-C, C=N or C-O and C=O bonds, respectively. The high-resolution XPS spectrum of O 1s (Figure 2E) was deconvoluted into two peaks with binding energies of 530.9 eV and 529.0 eV, corresponding to the C-O and C=O bonds, respectively. The crystal structure characterized by XRD (Figure 2F) revealed that TpPa-1 COFs had three diffraction peaks (from left to right) corresponding to the three planes (100, 110 and 001) of crystal. The above characterization demonstrated that the synthesized powder material is TpPa-1 COFs. The particle size distribution of TpPa-1 COFs is mainly in the range of 400–1000 nm, and its average size is about 738nm (Figure 2G).

### 3.2. Characterization of TpPa-1 COF/CA Membranes

**Figure 3:**
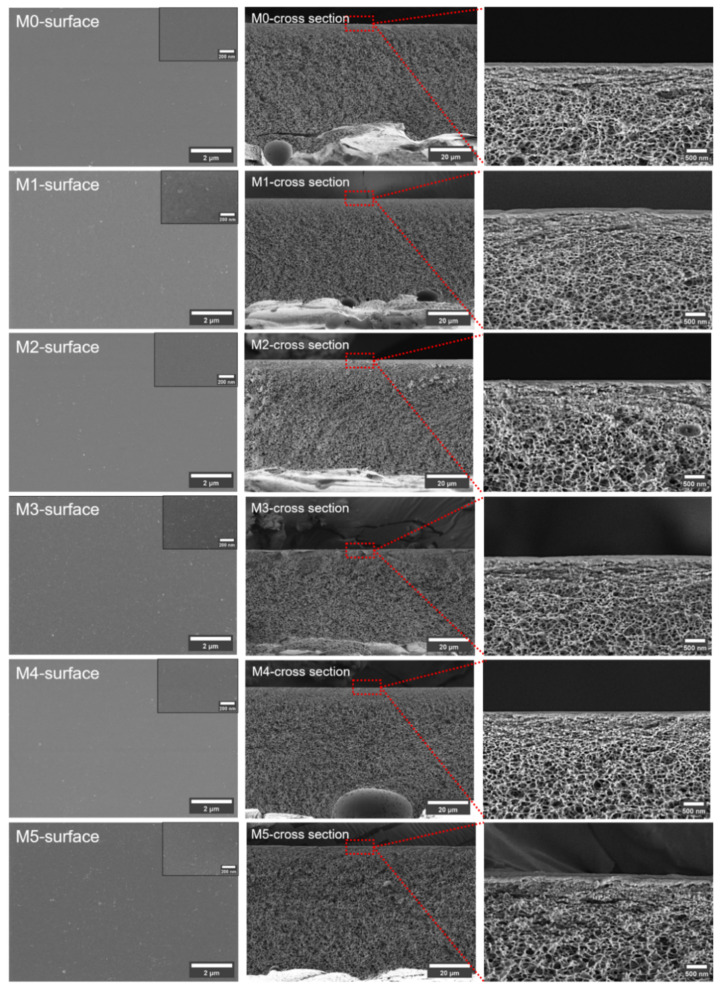
The SEM images of prepared membranes. (Red squares and straight lines represent the position of the third column of pictures in the membranes).

Figure 3 shows the membrane surfaces and cross sections. All the membranes have compact surface layers and porous spongy symmetrical structures. The formation of the spongy structure was a result of the excess Ac in the solvent used for the membrane preparation. This uniform morphology is beneficial in distinguishing the influence of the TpPa-1 COF nanomaterials on the membrane structure. According to the figures presented, the addition of TpPa-1 COFs did not have any intuitive effect on the membrane structure.

**Figure 4:**
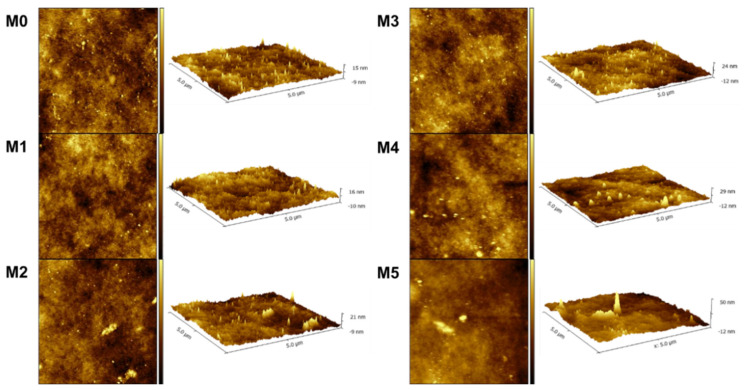
The AFM images of prepared membranes.

The surface roughness of the membranes was examined by AFM. The scanned pictures in 2D/3D of M0-M5 are shown in Figure 4. According to the scanned images, the mean roughness (Ra) and root mean-square roughness (Rq) were calculated and are presented in Table 2. The obtained data show that the surface of the membranes became rougher with the increase in the concentration of TpPa-1 COFs. This signifies that more membrane surface area could be contacted with water, which is beneficial for water transport [47]. Meanwhile, the increase in the roughness parameters also showed that TpPa-1 COFs were successfully incorporated into the membrane formulation.

**Figure 5:**
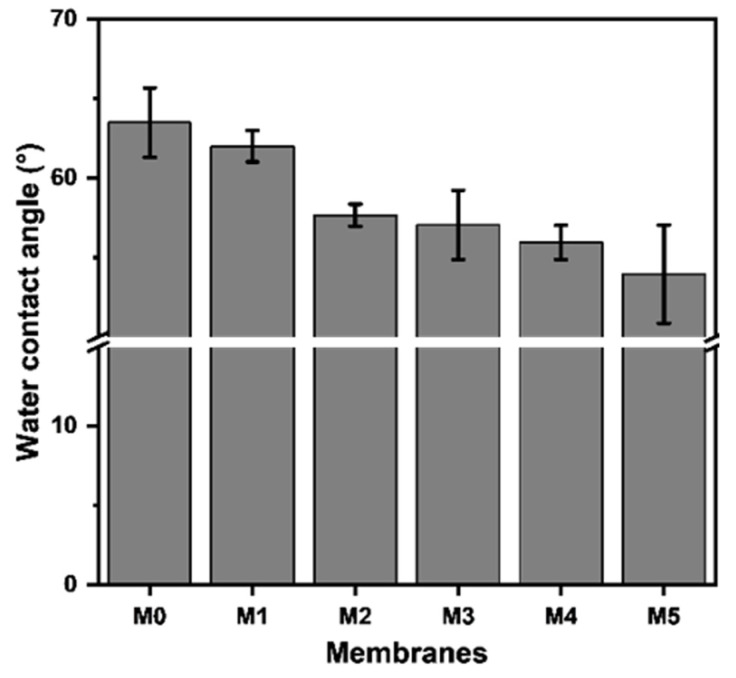
The water contact angle of the prepared membranes.

The water contact angle (WCA) symbolizing the hydrophilicity of membranes was analyzed and the results are presented in Figure 5. It can be seen that with the increase in TpPa-1 COF addition, the WCA of membranes steadily decreased from 63.5° to around 54.0°, which implies that the addition of the TpPa-1 COFs increased the hydrophilicity of M1-M5. The surface of TpPa-1 COFs contains hydrophilic groups of -NH_2_ and -OH, which may facilitate the water wettability of all the modified membranes (M1-M5).

**Figure 6:**
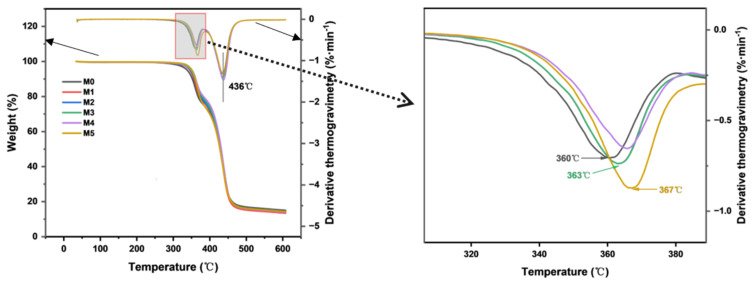
The thermogravimetric analysis and derivative thermogravimetry of M0–M5.

The effect of TpPa-1 COF addition on the thermal stability of the CA membrane was evaluated by TGA. As COFs can withstand heat of about 300–500 °C [14], the addition of TpPa-1 COFs is therefore expected to enhance the thermal stability of membranes. The results are presented in Figure 6. The thermal degradation temperatures of M0 and M1-M5 are similar, which was due to the addition of TpPa-1 COFs in a minute amount. The substantial mass loss of the CA membrane observed between 300 °C and 400 °C confirmed the decomposition of cellulose acetate. The distinct mass loss observed at 436 °C corresponds to the pyrolytic decomposition of non-woven fabric. The peak temperature of DTG represents the decomposition temperature. Compared with the decomposition temperature of 360 ℃ of M0, the decomposition temperature of the membranes containing TpPa-1 COFs still increases. When the addition of TpPa-1 COFs is 0.00025 wt%, the membrane is degraded at 367 °C, which shows that the thermal stability of modified membranes is further enhanced. This analysis also confirms that TpPa-1 COFs were successfully embedded in the membranes.

### 3.3. Separation Performance of Membranes

**Figure 7:**
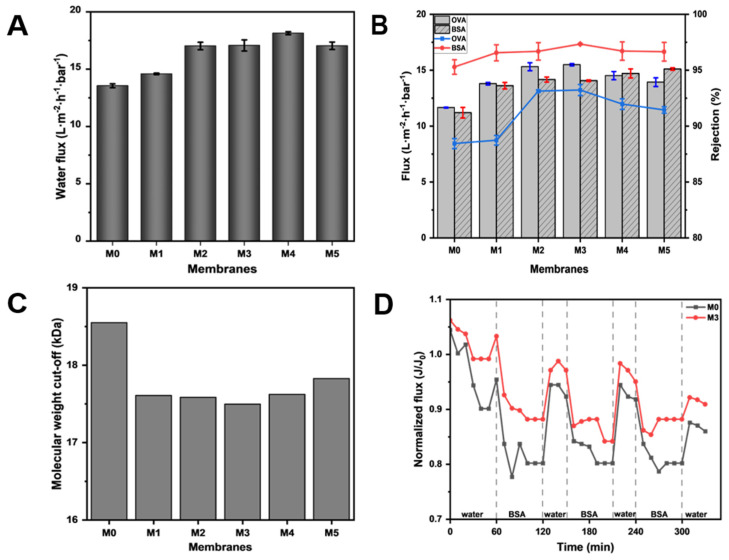
(**A**) The pure water flux of M0–M5; (**B**) the flux and protein rejection of M0–M5; (**C**) the molecular weight cut-off of M0–M5; (**D**) the anti-fouling performance of M0 and M3.

In order to clarify the effect of TpPa-1 COFs on the separation performance of modified membranes, the ultrafiltration performance of M0-M5 for the protein solution was studied. As can be seen from Figure 7A, with the addition of TpPa-1 COFs, the pure water flux of the membranes effectively increased. The pure water flux reached the maximum at M4, which is about 1.34 times that of M0. With the increase in the membrane surface roughness (Figure 4), the contact area of water was enlarged and water molecules could transport better on the membrane surface. The -OH and -NH_2_ of TpPa-1 COFs also enhanced the hydrophilicity of the membranes and promoted water transmission through the membranes. In addition, the porous crystal structure of TpPa-1 COFs provided more channels for the transmission of water molecules, which increased the water penetration. Furthermore, for water molecules passing through the membrane, the “gutter effect” formed by the nanoflower structure of TpPa-1 COFs may promote water transport efficiency [48]. However, when the concentration of TpPa-1 COFs is 0.00025 wt%, the pure water flux of M5 decreased slightly. The reason may be that redundant nanomaterials aggregate on the membrane structure, hence decreasing the number of nanochannels for water permeation. A similar observation was noted in another study reported elsewhere where COFs were used to boost permeation [49].

The flux and rejection of OVA and BSA solution also showed a similar tendency as that of pure water flux. The protein flux of modified membranes showed an advantage over the pristine CA membrane (M0) in Figure 7B. These results once again validate the influence of TpPa-1 COFs in improving the transport efficiency of water molecules in membranes. Meanwhile, it can be seen in Figure 7B that TpPa-1 COFs successfully increased the protein rejection of modified membranes. When the addition of TpPa-1 COFs increased from 0 wt% to 0.00015 wt%, the rejection of OVA increased from 88.44% to 93.23%, and the rejection of BSA increased from 95.29% to 97.34%. M3 has a smaller pore size than M0, which can effectively intercept more substances that are larger than the pore size of the membrane.

According to the rejection of PEG with different molecular weights, the MWCOs of the fabricated membranes were calculated and are shown in Figure 7C. The MWCO value of each membrane was consistent with its corresponding protein rejection ability, which shows that adding TpPa-1 COFs into CA membrane formation can effectively improve their rejection properties. The separation of the ultrafiltration membrane is largely based on physical screening; thus, the selectivity of the ultrafiltration membrane largely depends on the characteristics (pore size, pore distribution and porosity) of the membrane pore [50]. As the MWCO of membranes decreased from 18.5 kDa to 17.5 kDa, the corresponding effective pore diameter (μp) of membranes changed from 3.99 nm (M0) to 3.86 nm (M3). Therefore, the membrane with the smaller pore size will have better pollutant rejection performance.

The anti-fouling performance tests were conducted using 1 g·L^−1^ BSA solution to evaluate the stability and anti-fouling resistance performance of modified membranes. The experimental results (Figure 7D) show that the membrane flux cannot be completely recovered after water filtration due to the irreversibility of membrane fouling. Through experiments, the FRR of M3 can reach 90.9%, which is higher than M0 (86.0%), indicating that the anti-fouling resistance performance of M3 is better than M0.

### 3.4. Characterization of Protein Adsorption of Membranes

**Figure 8:**
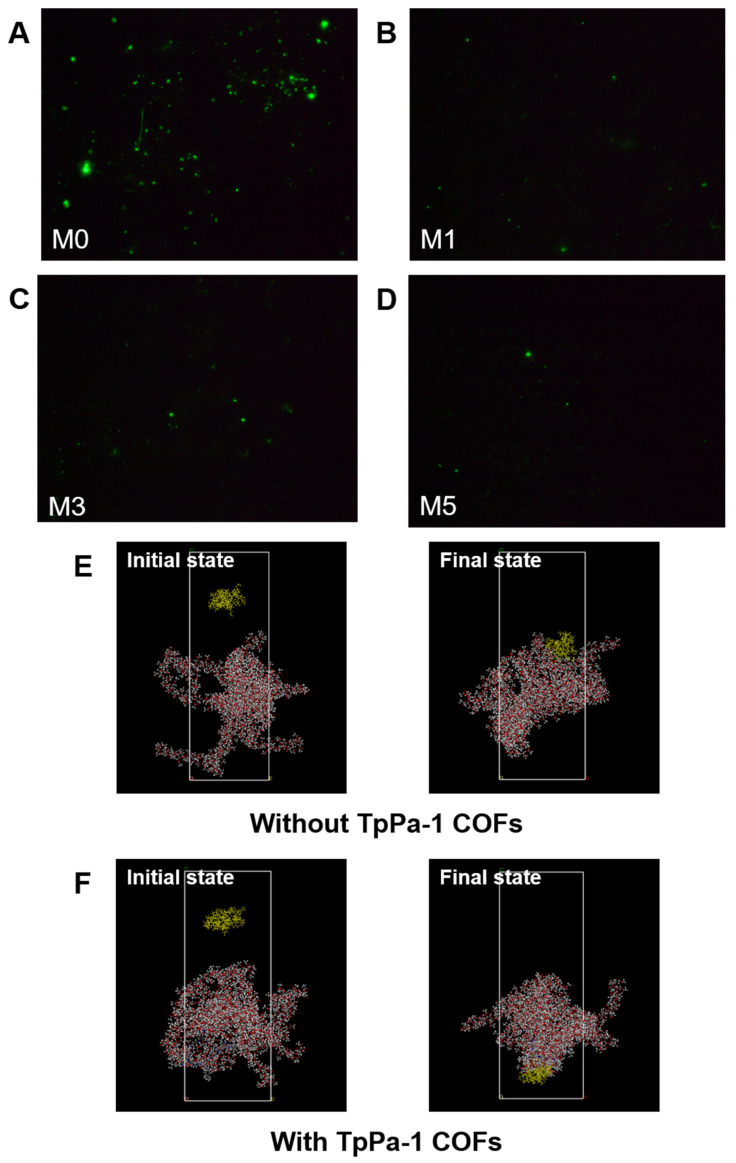

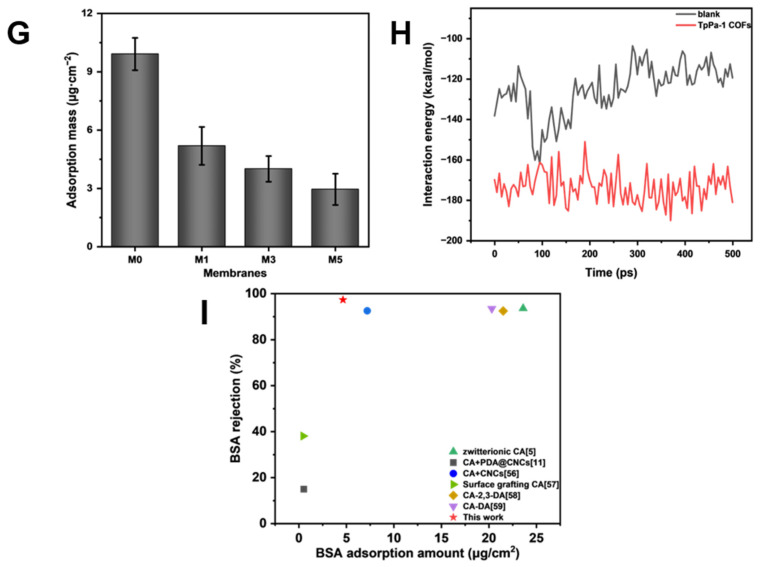
(**A**–**D**) The fluorescence images of the membrane surfaces; (**E**,**F**) simulation system for BSA adsorption of membranes without and with TpPa-1 COFs: BSA molecule, yellow; C, gray; O, red; N, blue; H, white; (**G**) the protein adsorption mass of membranes; (**H**) the interaction energy between membranes and BSA; (**I**) the comparison of TpPa-1 COF/CA membranes and other CA membranes.

As shown in Figure 8G, the amount of protein adsorption on the surface of CA membranes decreased with an increase in TpPa-1 COF concentration, and the BSA adsorption mass also became smaller with an increase in TpPa-1 COFs. The BSA adsorption mass of the membranes decreased from a maximum of 10.87 μg·cm^−2^ for M0 to 3.81 μg·cm^−2^ for M5. The fluorescence images of BSA on the membrane surfaces further confirmed the effect of TpPa-1 COFs on reducing the protein adsorption of CA membranes (Figure 8A–D). Compared with the synthetic polymer membrane, the natural polymer CA membrane has less protein adsorption [51,52], and the addition of TpPa-1 COFs enlarges this advantage. The reason may be that the hydrophilicity of the modified membranes is enhanced, hence, water molecules combine with the membrane surface to form a hydration boundary layer, which hinders the adhesion of BSA on the membrane surface [53,54].

MD simulations were applied to assess the interaction energy between BSA and CA membranes, for exploring the enhanced protein adsorption resistance of TpPa-1 COF-modified membranes. The adsorption of BSA with/without TpPa-1 COFs can be analyzed by MD simulations from a microscopic point of view. The initial state and final state of the two systems (with/without TpPa-1 COFs) in the MD simulation process are as shown in Figure 8E,F. The interaction energy between blank CA/TpPa-1 COF membranes and BSA is shown in Figure 8H. The interaction energies calculated are coulombic in nature. The interaction between blank CA/TpPa-1 COF membranes and BSA is repulsive in nature. The average interaction energy between the modified CA membrane and BSA was -173.58 kcal/mol, which is higher in absolute value compared with that of the pristine CA membranes and BSA (−125.28 kcal/mol). Due to the enhancement of the interaction energy between the modified CA membrane and BSA, BSA was repelled away from the membrane better [55]. This shows that the addition of TpPa-1 COFs enhanced efficaciously the protein adsorption resistance of CA membranes. In addition, the enhancement of the interaction energy is also beneficial to the membranes’ rejection effect.

When compared with the existing CA membrane research literature [5,11,56,57,58,59], TpPa-1 COF/CA membranes show advantages in terms of rejection rate and protein adsorption resistance (Figure 8I). In addition, the research on the protein adsorption of existing CA membranes and ultrafiltration membranes of other polymer materials are listed in detail in Table 3. Compared with previous studies, the incorporation of TpPa-1 COFs in CA mixed matrix membranes not only enhances their anti-protein adsorption properties but also achieves protein rejection rates comparable with or superior to those reported for reported ultrafiltration membranes. Although the M3 membrane does not exhibit high permeation flux relative to other reported ultrafiltration membranes, its low protein adsorption propensity renders it suitable for protein separation and purification applications, effectively minimizing protein loss during operational processes.

## 4. Conclusions

In this work, we synthesized TpPa-1COFs by the solvothermal method, and blended them into a CA membrane formulation to study the effect of TpPa-1 COF addition on the properties of the CA membrane. Compared with the pristine CA membrane, the surface roughness, hydrophilicity, pore size and thermal stability of the modified membranes were improved with the addition of TpPa-1 COFs. Furthermore, the proper addition of TpPa-1 COF concentration (in M3) could not only raise the pure water permeability of the CA membrane (~26.3% more than that of M0), but also simultaneously enhanced its rejection performance (with BSA rejection rising up to 97.34%), which successfully broke the permeability/selectivity trade-off in membrane applications. Furthermore, the addition of TpPa-1 COFs also reduced the protein adsorption of CA membranes, which minimized protein loss and slowed down membrane fouling. In conclusion, this study has demonstrated that TpPa-1 COFs as a nanomaterial modifier can enhance both the permeability and selectivity of the CA membrane, thus providing a promising prospect for improving the performance of ultrafiltration membranes.

## Figures and Tables

**Table 1:** The composition of casting solutions for TpPa-1 COF/CA membranes.

No.	CA (wt%)	DMAc/Ac (*w*/*w*)	TpPa-1 COFs (wt%)
		Total (78 wt%)	
M0	22	11/9	0
M1	22	11/9	0.000075
M2	22	11/9	0.0001
M3	22	11/9	0.00015
M4	22	11/9	0.0002
M5	22	11/9	0.00025

**Table 2:** The surface roughness parameters and WCA of M0-M5.

**No.**	**R_a_ (nm)**	**R_q_ (nm)**	**WCA (°)**
M0	1.95 ± 0.19	2.59 ± 0.27	63.50 ± 2.19
M1	2.08 ± 0.13	2.68 ± 0.17	62.02 ± 0.99
M2	2.17 ± 0.25	2.76 ± 0.25	57.68 ± 0.70
M3	2.24 ± 0.12	2.92 ± 0.04	57.06 ± 2.18
M4	2.55 ± 0.45	3.21 ± 0.42	55.96 ± 1.08
M5	2.66 ± 0.43	3.51 ± 0.52	53.96 ± 3.09

**Table 3:** Comparison results of different ultrafiltration membranes.

Polymer	Modification Method	Material	Permeance (L·m^−2^·h^−1^·bar^−1^)	BSA Rejection (%)	BSA Adsorption Amount (μg/cm^2^)	Ref.
zwitterionic CA	novel material	/	137.98	93.6	23.6	[5]
CA	mix	PDA@CNCs	320	12.4–15	0.52	[11]
PEN-COOH-60%	novel material	/	351.7	99.3	56.8	[51]
R-PI	/	/	88.32	96.3	112.7	[52]
Z-PI	novel material	/	127.65	93.4	43.8	[52]
CA	mix	CNCs	~40	92.55	7.194	[56]
CA	surface grafting	EPDMDAC	308	38.1	0.45	[57]
CA-2,3-DA	novel material	/	167.3	92.5	21.5	[58]
CA-DA	novel material	/	181.2	93.5	20.3	[59]
PVDF	surface grafting	zwitterion	~80	~70	~2.0	[60]
PVDF	mix	polyaniline	~100	~92	30	[61]
PVDF	mix	sulfonated polyaniline	~160	~90	3	[61]
CA (M3)	mix	TpPa-1 COFs	17.11	97.34	4.64	This work

## Data Availability

The original contributions presented in this study are included in the article. Further inquiries can be directed to the corresponding author(s).

## References

[1] Ulbricht M. (2006). Advanced functional polymer membranes. Polymer.

[2] Madaeni S.S. (1999). The application of membrane technology for water disinfection. Water Res..

[3] Rosberg R. (1997). Ultrafiltration (new technology), a viable cost-saving pretreatment for reverse osmosis and nanofiltration—A new approach to reduce costs. Desalination.

[4] Liu Y., Wang G. (2016). Membranes: Technology and Applications. Nanostructured Polymer Membranes.

[5] Liu Y., Huang H.T., Huo P.F., Gu J.Y. (2017). Exploration of zwitterionic cellulose acetate antifouling ultrafiltration membrane for bovine serum albumin (BSA) separation. Carbohyd. Polym..

[6] Mehta A., Zydney A.L. (2005). Permeability and selectivity analysis for ultrafiltration membranes. J. Membr. Sci..

[7] Opong W.S., Zydney A.L. (1991). Diffusive and Convective Protein-Transport through Asymmetric Membranes. Aiche J..

[8] Susanto H., Ulbricht M. (2009). Characteristics, performance and stability of polyethersulfone ultrafiltration membranes prepared by phase separation method using different macromolecular additives. J. Membr. Sci..

[9] Zhu J.Y., Hou J.W., Uliana A., Zhang Y.T., Tian M.M., van der Bruggen B. (2018). The rapid emergence of two-dimensional nanomaterials for high-performance separation membranes. J. Mater. Chem..

[10] Dass L.A., Alhoshan M., Alam J., Muthumareeswaran M.R., Figoli A., Shukla A.K. (2017). Separation of proteins and antifouling properties of polyphenylsulfone based mixed matrix hollow fiber membranes. Sep. Purif. Technol..

[11] Yao A.R., Yan Y.Q., Tan L., Shi Y.D., Zhou M., Zhang Y., Zhu P.X., Huang S.J. (2021). Improvement of filtration and antifouling performance of cellulose acetate membrane reinforced by dopamine modified cellulose nanocrystals. J. Membr. Sci..

[12] Hong J.M., He Y. (2014). Polyvinylidene fluoride ultrafiltration membrane blended with nano-ZnO particle for photo-catalysis self-cleaning. Desalination.

[13] Machado T.F., Serra M.E.S., Murtinho D., Valente A.J.M., Naushad M. (2021). Covalent organic frameworks: Synthesis, properties and applications-an overview. Polymers.

[14] Martín-Illán J.A., Rodríguez-San-Miguel D., Zamora F. (2023). Evolution of covalent organic frameworks: From design to real-world applications. Coordin. Chem. Rev..

[15] Côté A.P., Benin A.I., Ockwig N.W., O’Keeffe M., Matzger A.J., Yaghi O.M. (2005). Porous, crystalline, covalent organic frameworks. Science.

[16] Feng X., Ding X.S., Jiang D.L. (2012). Covalent organic frameworks. Chem. Soc. Rev..

[17] Ding S.Y., Wang W. (2013). Covalent organic frameworks (COFs): From design to applications. Chem. Soc. Rev..

[18] Geng K.Y., He T., Liu R.Y., Dalapati S., Tan K.T., Li Z.P., Tao S.S., Gong Y.F., Jiang Q.H., Jiang D.L. (2020). Covalent organic frameworks: Design, synthesis, and functions. Chem. Rev..

[19] Jaid G.M., Abdulrazak A.A., Meskher H., Al-Saadi S., Alsalhy Q.F. (2024). Metal-organic frameworks (MOFs), covalent organic frameworks (COFs), and hydrogen-bonded organic frameworks (HOFs) in mixed matrix membranes. Mater. Today. Sustain..

[20] Song Y.P., Sun Q., Aguila B., Ma S.Q. (2019). Opportunities of covalent organic frameworks for advanced applications. Adv. Sci..

[21] Meng W.Q., Xue Q., Zhu J.Y., Zhang K.S. (2024). Exploiting sulfonated covalent organic frameworks to fabricate long-lasting stability and chlorine-resistant thin-film nanocomposite nanofiltration membrane. Npj. Clean. Water..

[22] Meng W.Q., Zhu J.Y., Xue Q., Zhang K.S. (2023). Incorporating imine-based covalent organic frameworks nanosheet as an active filler for long-term nanofiltration desalination. J. Membr. Sci..

[23] Zhang M.M., Liu Z.W., Yin Z.Y., Yang C., Long M.Y., Lyu B., You X.D., Zhang R.N., Wu H., Cheng L.J. (2024). Anionic COF nanosheets construct porous and charged interlayer for the preparation of high-performance desalination membranes. J. Membr. Sci..

[24] Zhao J.Y., Wang S.W., Pan F.S., Zhang H., Zhang S., Jiang Z.Y. (2024). Leaf-inspired hybrid membrane assembled by covalent organic framework and cellulose nanocrystals for efficient desalination. J. Membr. Sci..

[25] Lin X.G., He Y.S., Zhang Y.T., Yu W.Y., Lian T. (2021). Sulfonated covalent organic frameworks (COFs) incorporated cellulose triacetate/cellulose acetate (CTA/CA)-based mixed matrix membranes for forward osmosis. J. Membr. Sci..

[26] Luo H., Bai X.P., Liu H.X., Qiu X., Chen J.Q., Ji Y.B. (2022). β-Cyclodextrin covalent organic framework modified-cellulose acetate membranes for enantioseparation of chiral drugs. Sep. Purif. Technol..

[27] Duong P.H.H., Kuehl V.A., Mastorovich B., Hoberg J.O., Parkinson B.A., Li-Oakey K.D. (2019). Carboxyl-functionalized covalent organic framework as a two-dimensional nanofiller for mixed-matrix ultrafiltration membranes. J. Membr. Sci..

[28] Ma Y.F., Yuan F., Yu Y., Zhou Y.L., Zhang X.X. (2020). Synthesis of a pH-responsive functional covalent organic framework via facile and rapid one-step postsynthetic modification and its application in highly efficient N^1^-methyladenosine extraction. Anal. Chem..

[29] Chandra S., Kandambeth S., Biswal B.P., Lukose B., Kunjir S.M., Chaudhary M., Babarao R., Heine T., Banerjee R. (2013). Chemically Stable Multilayered Covalent Organic Nanosheets from Covalent Organic Frameworks via Mechanical Delamination. J. Am. Chem. Soc..

[30] Kandambeth S., Mallick A., Lukose B., Mane M.V., Heine T., Banerjee R. (2012). Construction of Crystalline 2D Covalent Organic Frameworks with Remarkable Chemical (Acid/Base) Stability via a Combined Reversible and Irreversible Route. J. Am. Chem. Soc..

[31] Shende P., Kasture P., Gaud R.S. (2018). Nanoflowers: The future trend of nanotechnology for multi-applications. Artif. Cell Nanomed. B.

[32] Lee S.W., Cheon S.A., Kim M.I., Park T.J. (2015). Organic-inorganic hybrid nanoflowers: Types, characteristics, and future prospects. J Nanobiotechnol.

[33] Lin J.K., Fu C.L., Zeng W.C., Wang D., Huang F., Lin S., Cao S.L., Chen L.H., Ni Y.H., Huang L.L. (2023). Regulating the structure of cellulose-based ultrafiltration membrane to improve its performance for water purification. Ind. Crop. Prod..

[34] Liu Y.W., Ahmed S., Sameen D.E., Wang Y., Lu R., Dai J.W., Li S.Q., Qin W. (2021). A review of cellulose and its derivatives in biopolymer-based for food packaging application. Trends. Food Sci. Technol..

[35] Vatanpour V., Pasaoglu M.E., Barzegar H., Teber O.O., Kaya R., Bastug M., Khataee A., Koyuncu I. (2022). Cellulose acetate in fabrication of polymeric membranes: A review. Chemosphere.

[36] Islam M.D., Uddin F.J., Rashid T.U., Shahruzzaman M. (2023). Cellulose acetate-based membrane for wastewater treatment-A state-of-the-art review. Mater. Adv..

[37] Li W.R., Wang Q., Cui F.Z., Jiang G.F. (2022). Covalent organic framework with sulfonic acid functional groups for visible light-driven CO_2_ reduction. RSC Adv..

[38] Arkhangelsky E., Duek A., Gitis V. (2012). Maximal pore size in UF membranes. J. Membr. Sci..

[39] Singh S., Khulbe K.C., Matsuura T., Ramamurthy P. (1998). Membrane characterization by solute transport and atomic force microscopy. J. Membr. Sci..

[40] Akkermans R.L.C., Spenley N.A., Robertson S.H. (2021). COMPASS III: Automated fitting workflows and extension to ionic liquids. Mol. Simulat..

[41] Toumi K.H., Bergaoui M., Khalfaoui M., Benguerba Y., Erto A., Dotto G.L., Amrane A., Nacef S., Ernst B. (2018). Computational study of acid blue 80 dye adsorption on low cost agricultural Algerian olive cake waste: Statistical mechanics and molecular dynamic simulations. J. Mol. Liq..

[42] Knani D., Barkay-Olami H., Alperstein D., Zilberman M. (2017). Simulation of novel soy protein-based systems for tissue regeneration applications. Polym. Adv. Technol..

[43] Salahshoori I., Montazeri N., Yazdanbakhsh A., Golriz M., Farhadniya R., Khonakdar H.A. (2023). Insights into the Adsorption Properties of Mixed Matrix Membranes (Pebax 1657-g-Chitosan-PVDF-Bovine Serum Albumin@ZIF-CO_3_-1) for the Antiviral COVID-19 Treatment Drugs Remdesivir and Nirmatrelvir: An In Silico Study. ACS Appl. Mater. Interfaces.

[44] Matsuba R., Kubota H., Matubayasi N. (2022). All-atom molecular simulation study of cellulose acetate: Amorphous structure and the dissolution of small molecule. Cellulose.

[45] Wu X., Hao P., He F., Yao Z., Zhang X. (2020). Molecular dynamics simulations of BSA absorptions on pure and formate-contaminated rutile (110) surface. Appl. Surf. Sci..

[46] Zhang Y.F., Fang T.M., Hou Q.G., Li Z., Yan Y.G. (2020). Water desalination of a new three-dimensional covalent organic framework: A molecular dynamics simulation study. Phys. Chem. Chem. Phys..

[47] Wong P.C.Y., Kwon Y.N., Criddle C.S. (2009). Use of atomic force microscopy and fractal geometry to characterize the roughness of nano-, micro-, and ultrafiltration membranes. J. Membr. Sci..

[48] Xu Y.C., Zhang W.T., Li Z.W., Shen L.G., Li R.J., Zhang M.J., Jiao Y., Lin H.J., Tang C.Y.Y. (2022). Enhanced water permeability in nanofiltration membranes using 3D accordion-like MXene particles with random orientation of 2D nanochannels. J. Mater. Chem..

[49] Yang G.H., Zhang Z., Yin C.C., Shi X.S., Wang Y. (2022). Boosting the permeation of ultrafiltration membranes by covalent organic frameworks nanofillers: Nanofibers doing better than nanoparticles. J. Membr. Sci..

[50] Fane A.G., Tang C.Y., Wang R., Wilderer P. (2011). 4.11—Membrane Technology for Water: Microfiltration, Ultrafiltration, Nanofiltration, and Reverse Osmosis. Treatise on Water Science.

[51] Wang Q., Zhang S.Y., Dai F.N., Yan X.Y., Qian G.T., Chen C.H., Yu Y.H. (2021). Enhanced antifouling property of poly(aryl ether nitrile) ultrafiltration membranes via copolymerization with phenolphthalin. J. Environ. Chem. Eng..

[52] Huang H.T., Yu J.Y., Guo H.X., Shen Y.B., Yang F., Wang H., Liu R., Liu Y. (2018). Improved antifouling performance of ultrafiltration membrane via preparing novel zwitterionic polyimide. Appl. Surf. Sci..

[53] Kumar R., Ismail A.F. (2015). Fouling control on microfiltration/ultrafiltration membranes: Effects of morphology, hydrophilicity, and charge. J. Appl. Polym. Sci..

[54] Chen S.F., Li L.Y., Zhao C., Zheng J. (2010). Surface hydration: Principles and applications toward low-fouling/nonfouling biomaterials. Polymer.

[55] Tiwari S., Gogoi A., Reddy K.A. (2021). Effect of an ionic environment on membrane fouling: A molecular dynamics study. Phys. Chem. Chem. Phys..

[56] Lv J.L., Zhang G.Q., Zhang H.M., Yang F.L. (2017). Exploration of permeability and antifouling performance on modified cellulose acetate ultrafiltration membrane with cellulose nanocrystals. Carbohyd. Polym..

[57] Zhou Y., Jiang Y.Z., Zhang Y., Tan L. (2022). Improvement of antibacterial and antifouling properties of a cellulose acetate membrane by surface grafting quaternary ammonium salt. ACS Appl. Mater. Interfaces.

[58] Ma X., Wang C.Y., Guo H.X., Wang Z.F., Sun N., Huo P.F., Gu J.Y., Liu Y. (2022). Novel dopamine-modified cellulose acetate ultrafiltration membranes with improved separation and antifouling performances. J. Mater. Sci..

[59] Guo H.X., Peng Y., Liu Y., Wang Z.F., Hu J.W., Liu J.H., Ding Q., Gu J.Y. (2020). Development and investigation of novel antifouling cellulose acetate ultrafiltration membrane based on dopamine modification. Int. J. Biol. Macromol..

[60] Chiang Y.C., Chang Y., Higuchi A., Chen W.Y., Ruaan R.C. (2009). Sulfobetaine-grafted poly(vinylidene fluoride) ultrafiltration membranes exhibit excellent antifouling property. J. Membr. Sci..

[61] Feng Q., Shen X., He Y., Zhao Y., Yan F., Chen L. (2017). Effect of Cross-linking on Antifouling Properties for PVDF-g-PACMO Membranes. J. Mater. Eng..

